# Interdisciplinary Perspectives on Patient Adherence to Therapy: Findings from a Qualitative Study

**DOI:** 10.3390/healthcare14142225

**Published:** 2026-07-22

**Authors:** Paula González-García, Lourdes Fernández-Seguín, Elena Escamilla-Martínez, Elena Fernández-García, Celia Gago-Valle, María Isabel García-Bernal

**Affiliations:** 1 Physiotherapy Department, Faculty of Nursing, Physiotherapy and Podiatry, Universidad de Sevilla, 41009 Seville, Spain; pgonzalez@us.es (P.G.-G.); lfdez@us.es (L.F.-S.); ibernal@us.es (M.I.G.-B.); 2 Instituto de Biomedicina de Sevilla-IBiS, Hospitales Universitarios Virgen del Rocío y Macarena/CSIC/Universidad de Sevilla, 41013 Seville, Spain; 3 CTS 1043: Salud, Fisioterapia y Actividad Física Research Group, Universidad de Sevilla, 41009 Seville, Spain; 4 CTS 1110: Understanding Movement and Self in Health from Science (UMSS) Research Group, Universidad de Sevilla, 41009 Seville, Spain; 5 Nursing Department, Centro Universitario de Plasencia, Universidad de Extremadura, 10600 Plasencia, Spain; escaelen@unex.es; 6 Nursing Department, Faculty of Nursing, Physiotherapy and Podiatry, Universidad de Sevilla, 41009 Seville, Spain; 7 CTS 1141: Clinical Research Applied to Care and New Care Paradigms (ICCAPA) Research Group, Instituto de Biomedicina de Sevilla-IBiS, Universidad de Sevilla, 41009 Seville, Spain; 8 Centro de Psicología Cristina Galbarro, 41005 Seville, Spain; c.gago@cristinagalbarro.es

**Keywords:** healthcare professionals, interdisciplinary collaboration, qualitative research, patient resilience, patient-centered care, therapeutic adherence

## Abstract

**Highlights:**

**What are the main findings?**

- Healthcare professionals identified patient-related factors, particularly motivation, health literacy, and beliefs about treatment, as commonly influencing therapeutic adherence, together with the quality of the therapeutic relationship.
- Discipline-specific adherence-promotion strategies were identified: nurses prioritize the teach-back technique and visual aids; physiotherapists leverage functional goal-setting and group dynamics; podiatrists use objective visual evidence; and psychologists focus on expectation-setting and patient autonomy.

**What are the implications of the main findings?**

- The complementary nature of discipline-specific adherence strategies highlights the value of structured interdisciplinary collaboration and cross-disciplinary learning to deliver consistent, coordinated adherence support to patients with chronic conditions.
- Resilience emerged as a transversal mediating factor across all four disciplines, supporting the integration of resilience-informed approaches, including positive reinforcement, shared decision-making, and psychoeducation, into routine clinical practice and professional training curricula.

**Abstract:**

**Background/Objectives:** Therapeutic adherence remains a critical challenge in the management of chronic conditions, yet most research has focused on pharmacological adherence from the perspective of physicians and pharmacists. The views of non-physician healthcare professionals—particularly physiotherapists, podiatrists, psychologists, and nurses—remain underrepresented. This study aims to explore how these four healthcare disciplines perceive the factors influencing therapeutic adherence, the strategies they use to support it, and how they address patient resilience in clinical practice. **Methods:** An exploratory qualitative study was conducted using semi-structured interviews with 32 healthcare professionals (8 per discipline) recruited through purposive sampling across diverse care settings across Spain. Data were analyzed using reflexive thematic analysis following a hybrid deductive–inductive approach. Quality criteria were applied in accordance with the standards proposed by Lincoln and Guba. **Results:** Three thematic domains emerged. Patient-related factors, especially motivation, health literacy, illness beliefs, and socioeconomic constraints, emerged as central determinants across all disciplines. Health system barriers, particularly limited consultation time and fragmented continuity of care, also constituted significant barriers. Discipline-specific strategies ranged from the teach-back technique and customized visual aids in nursing and podiatry to functional goal-setting and group-based dynamics in physiotherapy and expectation-setting and digital follow-up in psychology. Resilience was consistently described as a key mediating factor enabling patients to sustain adherence despite adversity. **Conclusions:** Therapeutic adherence is a multifactorial and discipline-sensitive phenomenon. The complementary strategies identified across professions support the value of coordinated interdisciplinary approaches. Resilience-informed interventions and profession-specific adherence training represent promising directions for improving clinical practice and professional education.

## 1. Introduction

Therapeutic adherence remains a critical challenge for effective chronic disease management and the sustainability of healthcare systems. It is broadly defined as the extent to which a patient’s behavior corresponds with agreed recommendations from a healthcare provider. Adherence encompasses not only medication intake but also lifestyle modifications, rehabilitation exercises, and participation in diverse therapeutic interventions [1] and is increasingly recognized as a multidisciplinary concern extending well beyond the pharmacological domain. Despite advances in treatment options, non-adherence remains highly prevalent and is consistently associated with poorer clinical outcomes, increased morbidity and mortality, and higher healthcare costs [2,3]. These consequences are particularly relevant in chronic conditions, where long-term self-management is essential, and healthcare systems increasingly emphasize patient-centered care and self-care models.

Adherence is widely recognized as a complex phenomenon influenced by interacting determinants at multiple levels, including patient-related factors, treatment characteristics, healthcare system features and socioeconomic determinants, including cost and social support [4,5]. Interventions targeting single factors often demonstrate limited effectiveness, whereas multicomponent and context-adapted approaches appear more promising [6]. Understanding this complexity demands qualitative inquiry capable of capturing the contextual influences and real-world experiences that shape adherence in everyday clinical practice.

Healthcare professionals play a central role in promoting adherence. They are involved in identifying barriers, providing therapeutic education, negotiating individualized treatment plans, and supporting follow-up. However, their ability to implement adherence-support strategies may be influenced by prior training, professional culture, and organizational constraints such as limited consultation time and high workload. Previous studies identified gaps in adherence-related training and emphasized the need for practical, context-sensitive tools to facilitate implementation in routine clinical practice [7]. Therefore, understanding how professionals perceive and address adherence in real-world settings is essential to bridge the gap between evidence and practice. Yet healthcare professionals are not a homogeneous group. Physiotherapists, podiatrists, psychologists, and nurses differ substantially in the nature of their therapeutic interventions, the frequency and continuity of patient contact, their clinical objectives, and the professional cultures that shape their reasoning [8]. These differences condition how each professional identifies adherence barriers and are likely to produce perceptions and priorities that diverge in clinically meaningful ways [9]. Recognizing such differences is not only relevant for understanding adherence as a phenomenon but also for designing complementary, discipline-informed interventions that capitalize on the specific strengths of each professional role [10,11].

Beyond the role of healthcare professionals, informal caregivers—particularly family members—constitute a critical component of the patient’s support system in the management of chronic conditions. Caregivers often facilitate treatment-related tasks, provide emotional support, and help patients navigate complex regimens, and their involvement has been associated with improved adherence outcomes [12]. However, the perspectives of healthcare professionals regarding how they engage with, and rely on, caregivers as adherence-supporting agents remain insufficiently explored, representing an additional gap this study seeks to partially address.

Patient resilience, defined as the capacity to cope with and recover from adversity, has emerged as a psychosocial factor with growing relevance for adherence. Evidence suggests that resilience may mediate the relationship between personal resources and self-care behaviors and is positively associated with adherence in chronic disease populations [13]. How healthcare professionals perceive, assess, and actively address resilience in their clinical encounters remains largely unexplored, representing a meaningful gap between the evidence base and everyday practice.

Despite the growing body of research on therapeutic adherence, several important gaps remain. The evidence base is dominated by quantitative studies and clinical trials focused predominantly on medication adherence, which capture adherence as a measurable outcome rather than as a lived, contextually embedded experience [14,15]. Qualitative studies examining professionals’ perspectives have focused largely on physicians, nurses, and pharmacists, leaving the viewpoints of non-physician healthcare professionals, particularly physiotherapists and podiatrists, substantially underrepresented [16]. Moreover, existing research has rarely examined how professionals from different disciplines compare in their perceptions of adherence barriers and strategies, despite the potential of such comparisons to reveal clinically meaningful differences and complementarities that could inform truly interdisciplinary interventions [10]. Finally, although patient resilience has been identified as a psychosocial mediator of adherence, how healthcare professionals perceive and address it in clinical practice remains largely unexplored [13,17]. Taken together, these gaps highlight the need for qualitative, interdisciplinary research that explores how therapeutic adherence is understood, negotiated, and addressed in real clinical contexts, drawing on the perspectives and experiences of healthcare professionals.

This study aims to explore how healthcare professionals perceive and interpret the factors influencing therapeutic adherence and the strategies they use to support it in clinical practice; to compare adherence-related perceptions and approaches; and to investigate how professionals perceive and address patient resilience within the adherence process. From an interpretive qualitative perspective, these objectives seek to generate interdisciplinary evidence that can inform multicomponent interventions, guide profession-specific training, and strengthen healthcare teams’ capacity to support adherence across diverse clinical settings.

## 2. Materials and Methods

### 2.1. Study Design

This study adopted an exploratory and descriptive qualitative design within an interpretivist epistemological framework, guided by Braun and Clarke’s reflexive thematic analysis [18,19]. From this perspective, knowledge of social phenomena, including clinical practice, is understood as context-dependent and shaped by the perspectives of those involved [20].

Semi-structured interviews were selected as the primary method of data collection. This method allows for in-depth and contextualized accounts while maintaining sufficient structure to enable comparison across participants and disciplines. The study is reported in accordance with the Reflexive Thematic Analysis Reporting Guidelines (RTARG) [21].

The research team consists of healthcare professionals from physiotherapy, podiatry, psychology, and nursing, all with more than five years of clinical experience, often working with patients with chronic conditions. The team shares the view that therapeutic adherence is central to treatment success. This assumption is acknowledged as a potential influence on both the formulation of the research questions and the interpretation of findings. In keeping with the interpretivist orientation of this study and the principles of reflexive thematic analysis, researcher assumptions were not viewed as sources of bias to be eliminated but as elements requiring ongoing reflexive consideration. Throughout the study, the research team critically reflected its preconceptions regarding treatment adherence and their potential influence on study design, data collection, and analysis. Regular discussions within the multidisciplinary team were used to challenge interpretations and promote reflexive awareness throughout the research process.

To reduce confirmatory bias, data coding and analysis were conducted by a researcher who was not involved in data collection. In addition, the research team engaged in ongoing critical discussion throughout the analytical process. Clinical experience was treated as a source of contextual insight rather than as a fixed interpretive framework [22].

### 2.2. Sampling Strategy and Participants

Participants were recruited through purposive sampling, with the aim of achieving maximum variation across key dimensions: professional discipline, care sector (public and private), care setting (primary care, hospital, and community), and geographical context (rural and urban). This approach was intended to capture a broad range of professional experiences related to therapeutic adherence, rather than to ensure statistical representativeness [23].

The study included professionals from four disciplines (physiotherapy, podiatry, psychology, and nursing). These professions were selected due to their close and sustained involvement in patient care and follow-up and their direct experience with treatment adherence. Inclusion criteria were current professional practice with at least one year of independent clinical experience; work in primary, hospital, community, or private care settings; and provision of written informed consent. Professionals in training without independent clinical practice, and those who did not consent to audio recording, were excluded.

According to Malterud et al. (2016) [24], sample size in qualitative research should be guided by the concept of information power: the more information the sample provides that is relevant to the study aim, the fewer participants are required. For this reason, a total of 32 to 40 interviews were planned, with a target of 8 to 10 participants per discipline. This range is consistent with qualitative research employing an exploratory design across multiple professional groups. The final sample size was determined by the richness, depth, and diversity of the data in relation to the study objectives. Richness was reflected in the detailed and information-rich accounts provided by participants, while depth was achieved through the exploration of contextual and underlying factors influencing treatment adherence. Diversity was ensured through the inclusion of professionals from different disciplines, care settings, sectors, geographical contexts, and levels of professional experience.

Recruitment remained flexible and was guided by the aim of capturing a wide range of perspectives across disciplines, rather than by a predefined notion of data saturation [25].

### 2.3. Recruitment Procedure

Participants were identified through professional associations, regulatory bodies, and professional networks across Spain. Recruitment was channeled through professional colleges and associations, which disseminated the invitation among registered members interested in participating, rather than through a facility-based sampling frame. Initial contact was made by email or telephone. Before the interviews, participants received a written information sheet describing the aims, procedures, and ethical considerations of the study. Written informed consent was obtained prior to data collection. Anonymity was ensured by replacing all identifying information with alphanumeric codes.

▪ *Interview Guide Development and Piloting*

The semi-structured interview guide was developed based on a systematic review of the literature on therapeutic adherence. It was organized into four thematic areas:

(1) factors influencing adherence related to the patient, treatment, healthcare system, and professional levels.
(2) perceptions of the relationship between patient resilience and adherence.
(3) strategies used to promote adherence and recommendations and
(4) experiences of success and difficulty in managing adherence.

A pilot interview was conducted to assess the clarity, flow, and comprehensiveness of the guide. Minor adjustments were made to the wording and sequence of questions where necessary.

### 2.4. Data Collection

Interviews were conducted by researchers from the same professional discipline as the participants, facilitating communication through a shared professional language and contextual understanding of clinical practice. To promote consistency across interviews, a common semi-structured interview guide was collaboratively developed by the multidisciplinary research team. A pilot interview was conducted prior to data collection to refine the guide and ensure the clarity and relevance of the questions. In addition, regular team meetings were held throughout the study to promote coherence in the interview process and a shared understanding of the study objectives.

To facilitate rapport and shared understanding, each interviewer conducted interviews exclusively with participants from the same discipline. Interviewers had no prior therapeutic relationship with participants. All interviews were audio-recorded with prior consent and subsequently transcribed verbatim.

### 2.5. Data Analysis

Data were analyzed using reflexive thematic analysis, following the approach proposed by Braun and Clarke [26]. A hybrid deductive–inductive strategy was adopted. Analysis was conducted manually to allow close engagement with the data. An initial analytic framework was developed based on literature. It included predefined categories related to the patient, treatment, healthcare system, and professional level, as well as strategies to promote adherence. The resilience domain, by contrast, was not assigned predefined codes; instead, two categories—perceived influence of resilience on adherence and observed differences in adherence according to patients’ level of resilience—emerged inductively during coding. These categories were used as a guiding framework rather than as a fixed structure, allowing new codes to emerge from the data.

The analysis proceeded in three stages: (1) initial line-by-line coding; (2) refinement and grouping of codes into categories; and (3) development of overarching themes.

Coding was carried out by a researcher who was not involved in data collection, with critical input from a second researcher to deepen and refine interpretations. This separation between data collection and analysis was maintained to reduce potential bias. The potential influence of professional discipline on the findings was also examined. A formal reflective journal was not maintained; reflexive engagement was instead conducted through ongoing discussion between researchers, which we acknowledge as a less formalized approach than a documented journal.

Numerical counts of codes by discipline are reported for descriptive and transparency purposes only and were not used as a criterion of thematic importance during analysis, consistent with reflexive thematic analysis principles. A theme matrix was developed to support the transition from codes to overarching themes.

A code co-occurrence network was generated using VOSviewer (version 1.6.21). Node size reflects code frequency and links represent code co-occurrence across participants.

### 2.6. Rigor and Trustworthiness

Several strategies were implemented to ensure the quality of the study, following the criteria proposed by Lincoln and Guba [20,27]. Credibility was supported through purposive sampling aimed at maximum variation, the use of discipline-matched interviewers, and the involvement of an independent researcher in the coding process. Dependability was ensured by maintaining a transparent and well-documented analytical procedure, including the use of an initial codebook and a structured, multi-phase coding process. Member checking was not undertaken; consistent with reflexive thematic analysis, themes are understood as the analyst’s interpretive account rather than facts to be verified by participants. Transferability was supported by providing detailed descriptions of the study context, participant characteristics, and analytical procedures, allowing readers to assess the applicability of the findings to other settings.

## 3. Results

### 3.1. Participant Characteristics

A total of 32 semi-structured interviews were conducted between February and March 2026, out of 35 professionals initially contacted (response rate: 91.4%); the most common reason for non-participation was lack of time. The mean interview duration was 24 min (range: 10–49 min).

Participant characteristics captured variation across key dimensions, discipline, care setting, geographical context, and sex, although with an uneven distribution in some categories, as detailed in Table 1 (see also Section 4.5). Participants reported working with a highly heterogeneous population, with age ranges spanning from pediatric groups to older adults, and included a substantial proportion of patients with chronic conditions (e.g., diabetes, oncology, pain), as well as vulnerable populations such as older, dependent, or socioeconomically disadvantaged individuals.

### 3.2. Interviews Analysis

The findings are presented across three thematic domains that reflect the study’s objectives and the distinct analytical register each requires. The first domain, factors influencing adherence, draws on participants’ perceptions of barriers and facilitators encountered in clinical practice and is reported descriptively and comparatively across disciplines. The second domain, strategies used to promote adherence, moves from perception to practice, examining how professionals actively respond to adherence challenges within their specific disciplinary contexts. The third domain, resilience and adherence, explores how professionals conceptualize and apply this construct; this section presents a more interpretive account of participants’ perspectives.

#### 3.2.1. Factors Influencing Adherence

**Figure 1:**
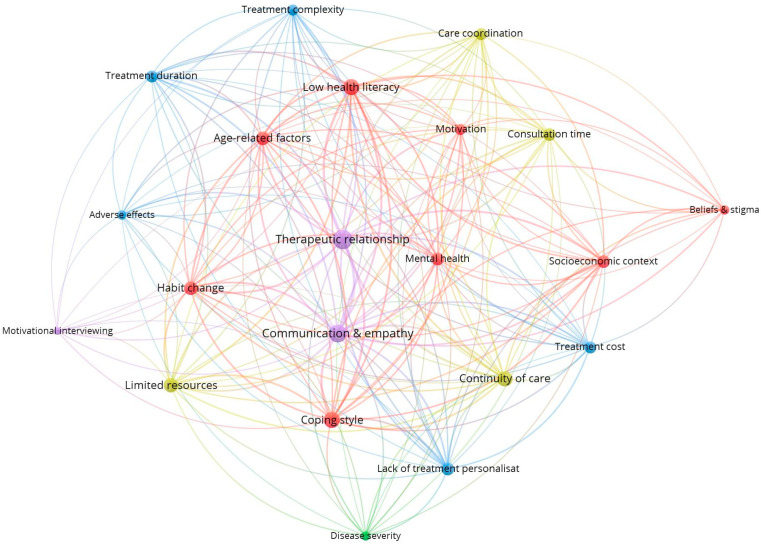
A code co-occurrence network of factors influencing treatment adherence. Each category is represented by a different color.

Figure 1 presents the co-occurrence network of the identified codes. Codes related to patient factors, treatment factors, healthcare system factors, and professional factors formed an interconnected network, highlighting the relationships between barriers and facilitators to adherence.

##### Patient-Related Factors

Patient-related factors emerged as a central theme across all four disciplines, encompassing motivation, health literacy, and socioeconomic context (Table 2). Participants commonly described **low motivation** and a tendency among patients to adopt a **passive role**, often delegating responsibility to the clinician. This was frequently linked to a perceived expectation of rapid results.

*“Today’s society looks for immediacy and is not willing to tolerate the suffering or the effort that a recovery process entails”*.

(PHY06)

**Limited health literacy** was identified as a key barrier across disciplines, as it affected patients’ ability to understand and sustain treatment. However, some physiotherapists emphasized that effective communication could mitigate these limitations.

*“The educational level is less relevant if the professional manages to adapt the explanations and the tasks to each patient’s level”*.

(PHY01)

**Beliefs, stigma, and shame** were also described as influencing adherence, with some variation across disciplines. While nurses and physiotherapists referred to deeply rooted beliefs that hinder engagement, psychologists highlighted mental health stigma as a specific barrier. **Mental health and other comorbidities**, including substance use, were frequently associated with reduced adherence. Podiatrists noted that patients managing multiple conditions often prioritized some treatments over others, while nurses described complex social situations in which adherence was particularly difficult to sustain.

*“A social stigma persists where psychologists are still not well-regarded by everyone”*.

(PSY05)

*“In complicated psychosocial environments or among drug users, despite health education, the personal situation makes it almost impossible to maintain long-term adherence”*.

(NUR06)

Participants described a range of **attitudes toward treatment**, including passivity, fear, fatalism, and differing levels of self-efficacy, highlighting the importance of fostering active engagement. Difficulty in changing established habits was particularly emphasized in podiatry, where adherence often depends on long-standing behaviors such as diet or smoking. **Socioeconomic constraints** and limited autonomy were also described as influencing adherence, particularly in long-term treatments and private care settings. Age was interpreted variably, sometimes as a facilitator (e.g., greater maturity) and sometimes as a barrier (e.g., physical limitations), although several participants noted that its influence depended on other factors such as motivation or family support.

*“Changes in deeply fixed habits are the most difficult to achieve, such as diet or tobacco consumption in diabetic patients”*.

(POD04)

##### Nature of the Health Condition

Only one code emerged regarding this matter: **the perceived severity of the disease**. In nursing and podiatry, the primary challenge to adherence is the lack of health awareness regarding asymptomatic conditions; conversely, a serious health event often serves as the most powerful catalyst for compliance. In the field of psychology, patient perception is closely tied to the cognitive need to “label” or give a name to their conditions. Physiotherapists and podiatrists further noted that clinical outcomes have a paradoxical effect on adherence: immediate results may lead to premature cessation of treatment, whereas delayed progress can similarly result in attrition.

*“An ulcer doesn’t hurt because sensibility is altered, so the patient is unaware of the danger until it is too late”*.

(POD03)

##### Treatment-Related Factors

Treatment-related factors were consistently identified as key determinants of adherence, although their meaning varied across disciplines. **Regimen complexity** was commonly mentioned. In nursing, this was primarily associated with polypharmacy and the management of multiple medications and schedules. In contrast, in physiotherapy and podiatry, complexity was linked to the effort required to perform exercises or maintain postural changes, with patients often preferring simpler or more passive interventions.

*“Patients prefer treatments that require less time and effort”*.

(POD08)

**Treatment duration** and the lack of immediate results were also commonly described as challenges. In physiotherapy and psychology, participants noted that patients often expect rapid improvement and may disengage from interventions requiring sustained effort over time. In nursing and podiatry, long-term treatments were associated with fatigue and loss of motivation, particularly in chronic conditions.

*“It also highlights the desperation of patients who seek ‘immediate relief’ under a biomedical model, expecting quick and effective solutions when psychotherapy requires time and has ups and downs”*.

(PSY06)

Perceived **adverse effects** differed by discipline. Nurses referred mainly to physical side effects (e.g., gastrointestinal or sexual symptoms) that could lead to treatment discontinuation, particularly when patients had not been adequately informed. In physiotherapy, fear of pain related to exercises or manual techniques was frequently mentioned, while in psychology, emotional discomfort associated with therapeutic processes was described as a barrier.

*“If the person feels that the pills make him feel unwell, he won’t take them”*.

(NUR03)

**Financial cost** was identified as an important barrier in private practice, particularly in podiatry and psychology, where access to treatment may be limited by patients’ economic resources. Across all disciplines, lack of personalization was described as negatively influencing adherence. Participants emphasized the importance of adapting treatment plans to patients’ individual circumstances, rather than expecting patients to conform to standardized protocols.

*“It is necessary to adapt the treatment to the patients’ needs and fears”*.

(PHY05)

##### Health System-Related Factors

Health system factors were described as important influences on adherence, particularly in relation to time constraints, continuity of care, and resource availability. **Limited consultation time** and high workload were frequently identified as barriers to effective communication and patient education, which participants considered essential for supporting adherence.

*“An adequate consultation time allows for listening and receiving feedback from the patient to verify if they have understood the information. If only one minute is available, you are only ‘putting out fires’”*.

(NUR08)

**Continuity of care** was especially emphasized in psychology, where participants highlighted the impact of staff turnover and short-term contracts on the therapeutic relationship. Repeated disruptions in care were described as discouraging for patients and associated with disengagement.

*“Short-term contracts and frequent staff changes make it difficult to build a lasting bond between the patient and us”*.

(PSY01)

Across disciplines, **institutional barriers and limited resources** were among the most frequently reported affecting adherence. Long waiting lists and service saturation were described as contributing to treatment delays and interruptions, particularly in physiotherapy and psychology. Nurses additionally noted that limited access to primary care may lead patients to rely on emergency services, while podiatrists referred to reduced availability of services within the public system.

**Interdisciplinary coordination and social support** were described as relevant for supporting adherence. Participants emphasized the importance of consistent communication across professionals and the need for coordinated care that reflects patients’ broader social context.

*“It is vital to have a clinical network where all doctors, nurses, and pharmacists deliver the same message”*.

(NUR07)

##### Professionals-Related Factors

The following two codes emerged as salient across all disciplines, reflecting shared professional concerns.

Across all disciplines, **communication and an empathic attitude** are viewed as vital tools to achieve adherence. Nurses emphasize that being a “visible” and accessible professional prevents patients from withholding information or lying about their compliance out of shame. Both physiotherapists and podiatrists highlight the necessity of using clear, non-technical language to ensure the patient truly understands their diagnosis.

*“You cannot talk using technical jargon, you have to use a language that the patient understands to eliminate the fear of the treatment”*.

(PHY02)

The **therapeutic relationship** and trust are described as the base of every intervention. In the field of psychology, this alliance is considered the factor that most explains clinical improvement, often more so than the specific technical model used. Establishing a secure bond is particularly crucial for vulnerable populations, such as migrants or trauma victims, to ensure they feel safe enough to speak freely. Similarly, in podiatry and nursing, a strong bond allows patients to openly share their difficulties with treatment, enabling the professional to provide better tools.

*“If there is not a therapeutic bond, your treatment will fail”*.

(PSY07)

While **communication skills** are sometimes seen as innate, some professionals argue that motivational interviewing is a form of “high technology” in primary care that must be specifically studied and practiced. These skills are essential for exploring the patient’s internal “motor” (their specific hobbies or interests) and using them to anchor the treatment plan.

### 3.2.2. Strategies to Promote Adherence

The adherence-promotion strategies across the four disciplines showed differences in how adherence was addressed in practice. While nursing and podiatry prioritize the verification of patient comprehension, physiotherapy and psychology focus on social motivation and the robustness of the therapeutic bond.

Table 3 shows all the strategies that emerged; however, the following section details the distinctive strategies employed by each discipline:

Nurses focus on health literacy and skills training, using the “Teach-back” technique, in which patients are asked to explain the treatment protocol as if they were the clinician. This ensures that technical information, particularly regarding complex pharmacological regimens, has been accurately internalized. Podiatrists also tend to use this technique. Additionally, nursing stands out for its use of family mediation and customized visual aids (e.g., manual infographics or medication diagrams for home display) to adapt intricate protocols to daily routines, especially of elderly patients or those with limited educational backgrounds.

*“When they arrive at ER, I make them like a quick check asking about their pills, when they take them and why”*.

(NUR01)

Physiotherapy mainly uses motivation through functional success and social dynamics. Physiotherapists utilize the consensus of functional goals as a primary strategy, aligning rehabilitative efforts with the patient’s personal desires (e.g., resuming pet care) to imbue physical exertion with vital meaning. A notable distinction is the strategic use of social and group components; in rehabilitative settings, peer-to-peer motivation is fostered to reduce absenteeism, occasionally extending to social gatherings that build a sense of “community” or “family.” In pediatric contexts, gamification and humor serve as the principal tools for securing child engagement.

*“For many elderly people, their time in physiotherapy is their social time, so they try not to miss it”*.

(PHY02)

In podiatry, visual evidence is used to counteract the lack of awareness associated with asymptomatic or painless podiatric pathologies (such as diabetic foot). These strategies rely on the constant reinforcement of information and the provision of objective technical evidence of progress. Technology is used not only for diagnosis but also to present patients with tangible data of their improvement, thereby bolstering resilience and consistency. Furthermore, in private practice, podiatrists employ economic management strategies, such as “fixed-price treatment packages,” to ensure patients complete the full clinical cycle.

*“I personally use the repetition: going over the instructions again and again”*.

(POD01)

In psychology, the therapeutic alliance and framing are core strategies. The fundamental strategy is not an external intervention, but the therapeutic bond and the alliance established during initial sessions. This discipline is characterized by the use of “clinical framing”, the clarification of expectations and confidentiality, and the strategic promotion of patient autonomy, transferring responsibility for the therapeutic process to the individual to activate intrinsic motivation. Additionally, psychology remains the discipline that most systematically utilizes digital reminders (e.g., SMS, WhatsApp, or specialized platforms) to mitigate appointment attrition in long-term follow-ups.

*“…reassure the patient that there won’t be moral statements and freedom to change therapist if he is not comfortable”*.

(PSY02)

**Figure 2:**
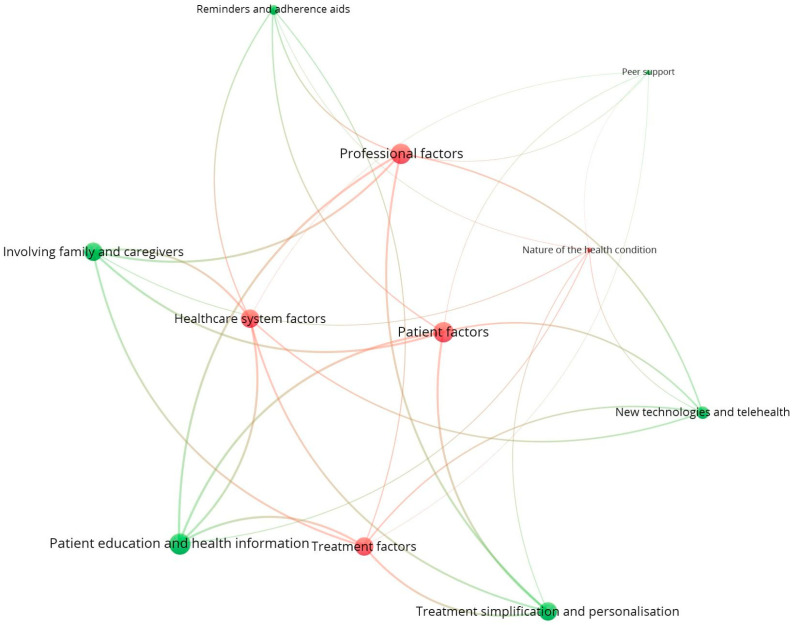
A network of factors influencing treatment adherence and strategies to promote it. Red color represents categories and green color strategies.

Figure 2 illustrates the network linking the categories of factors influencing treatment adherence with the consolidated intervention strategies identified in the interviews.

### 3.2.3. Resilience and Adherence

For the professionals interviewed, resilience was described as a fundamental and decisive mediating variable that directly influences a patient’s capacity to maintain therapeutic adherence. Most participants agree that a resilient patient adopts a proactive attitude toward recovery, helping patients continue treatment when facing challenges, adverse effects, or the absence of immediate results. Resilience is described as an adaptive “force” or capacity that facilitates consistency throughout protracted and demanding therapeutic processes.

*“… is the strength or the capacity that allows the patient to follow a treatment…”*.

(POD08)

In disciplines such as physiotherapy and podiatry, resilience is closely associated with effort capacity and the tolerance of necessary discomfort required for improvement. Interviewees observe that patients with higher levels of resilience, frequently older adults with a history of significant life challenges, demonstrate better acceptance of functional limitations and painful treatments, recognizing them as necessary steps toward long-term benefits. Conversely, a lack of resilience is linked to a “culture of immediacy,” where patients experience instant frustration if recovery is not instantaneous or effortless.

*“Resilience helps people to admit that a long-suffered condition might not improve that fast”*.

(PHY07)

From a psychological perspective, resilience allows the patient to use the therapeutic space as a source of support, rather than remaining in a more passive role, as described by participants. A highly resilient patient is capable of conceptualizing their pain and engaging with vulnerable themes in therapy without surrendering. Furthermore, in nursing, severe health events (such as a myocardial infarction or an HIV diagnosis) are identified as clinical triggers that drastically elevate both resilience and disease awareness, thereby improving compliance with prescribed guidelines.

*“…wrote me a letter thanking me for having, for the first time, a space where she could be listened to and cared for”*.

(PSY03)

However, a critical perspective also emerged regarding the risk of framing resilience as a “war metaphor.” Some professionals cautioned that by requiring a patient to be “strong,” there is a danger of inducing guilt if the individual fails to overcome their situation. Consequently, strategies to strengthen resilience do not rely on pressure; instead, they focus on positive reinforcement of incremental achievements, psychoeducation, shared decision-making, and the mobilization of the individual’s pre-existing strengths. Ultimately, resilience was described as supporting patients in integrating treatment into their lives despite adversity.

*“…on top of her illness, she felt guilty for not being strong enough”*.

(NUR07)

Family and home caregiver involvement was identified as an important facilitator of treatment adherence, particularly through caregiver training for dependent patients and family processes to resolve conflicts that may hinder adequate care.

*“The support of home caregivers often makes the difference between good and poor adherence”*.

(NUR01)

**Figure 3:**
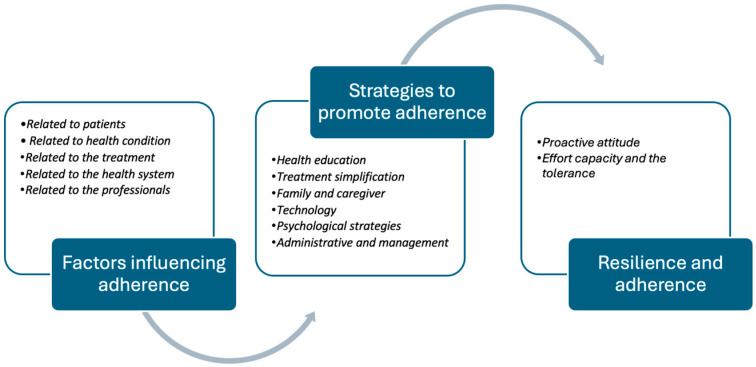
Multilevel factors influencing therapeutic adherence and discipline-specific strategies across nursing, physiotherapy, podiatry and psychology.

Overall, the findings indicate that therapeutic adherence is understood across disciplines as a multifactorial and patient-dependent process, shaped primarily by health literacy, treatment attitudes, home caregivers and the quality of the clinician–patient relationship. Although common barriers and facilitators were identified, discipline-specific interpretations and management approaches were evident, reflected in the range of strategies used, from educational and monitoring interventions to relational and motivational techniques. Notably, resilience emerged as a consistent determinant of patients’ ability to sustain adherence, particularly in long-term and demanding treatment contexts, as summarized in Figure 3.

## 4. Discussion

This study explored how healthcare professionals from four disciplines perceive and address therapeutic adherence in clinical practice, highlighting both shared and discipline-specific perspectives. Overall, adherence was described as a complex and multifactorial process, strongly shaped by patient-related factors, particularly health literacy and attitudes toward treatment and the quality of the relationship between the professional and the patient. While common barriers and facilitators were identified across disciplines, differences emerged in how professionals interpreted and managed these challenges in practice. Strategies to support adherence reflected these variations, ranging from educational and monitoring approaches to relational and motivational techniques. In addition, resilience was consistently described as an important factor influencing patients’ capacity to sustain adherence, particularly in the context of long-term and demanding treatments.

It should be noted that, in this sample, professional discipline was structurally associated with both care sector and years of clinical experience; some of the differences discussed below may therefore reflect these overlapping factors rather than discipline alone.

### 4.1. Factors Influencing Adherence

The analysis of the four healthcare disciplines integrated into this study reveals a consensus: the patient’s intrinsic factors represent the most significant variable in treatment compliance, given that the patient acts as the final link in the execution of the clinical decision. Although the healthcare system may guarantee access to treatment and the socioeconomic environment may be favorable, it is ultimately the individual’s psychological and cognitive dimension that turns a medical prescription into a sustained behavioral habit.

For instance, a lack of motivation and passive personal attitudes cause adherence to drop significantly if the patient does not believe in their capacity for action [28]. The desire for short-term results conflicts with the chronicity of diseases, where delayed gratification is a documented psychological barrier in behavioral models [29]. Furthermore, shifting all responsibility onto healthcare personnel nullifies the capacity for empowerment, which is a predictive factor for therapeutic success [30].

Communication barriers and technical language create a health literacy gap that correlates with medication errors [31]. The socioeconomic context is confirmed as a critical social determinant, as precariousness limits actual access despite the patient’s intention [32]. Finally, age shows a complex relationship. Some studies show that younger individuals present lower adherence due to lax lifestyles, whereas in older adults, polypharmacy and cognitive impairment are the limiting factors [33].

In our study, we have also observed controversy in this regard; while maturity can be a positive factor, the dependency and family support required in advanced age act as limiting factors. The combination of these intrinsic individual variables directly conditions their perception of the disease. In this sense, the importance that the patient assigns to therapeutic compliance is usually proportional to their sense of vulnerability; thus, ‘severity’ goes beyond objective clinical data and becomes a subjective construct. Therefore, healthcare personnel should intervene through patient education, helping align the patient’s interpretation with clinical reality to improve adherence [34].

Adherence is compromised when physical effort and pain aversion emerge as obstacles that exceed the individual’s self-efficacy. Persistence breaks down if the therapeutic burden is perceived as unmanageable [35]. When faced with a severe diagnosis, patients weigh attitudes toward discomfort and physical effort more heavily than clinical severity itself when deciding whether to continue treatment. This reflects a temporal discounting pattern, where the immediate gratification of avoiding suffering is prioritized over the expectation of long-term recovery. Ultimately, the perception of lacking the resources to cope neutralizes the intention to follow the established clinical guidelines [36].

Systemic deficiencies, such as delays in waiting times and fragmentation of care, act as structural barriers that ultimately undermine the patient’s motivation, turning the therapeutic burden into something unmanageable. The lack of stable communication, derived from high professional turnover, causes a collapse in self-efficacy, as the system fails to consolidate the trust required to manage pain or adverse effects [37].

Despite the obstacles, the healthcare professional possesses a decisive capacity for influence over multiple factors that favor therapeutic adherence, a point where all disciplines analyzed in this study converge. An interaction based on empathy and communicative clarity strengthens the therapeutic alliance, mitigating the uncertainty and fear that typically compromise treatment continuity. This bond allows the patient to verbalize concerns regarding pain or effort, facilitating the clinician’s ability to readjust the perception of necessity against individual barriers. By feeling supported by a system often perceived as overwhelmed, the subject develops a resilient trust that protects against the impact of side effects. Ultimately, this humanized connection succeeds in converting a clinical prescription into a shared decision, substantially increasing the persistence and efficacy of the therapeutic process [38].

### 4.2. Strategies to Promote Adherence

Regarding strategies to promote adherence, several approaches emerge, including verification of patient comprehension, social motivation, and the strength of the therapeutic relationship. The findings of the present study are consistent with previous literature, which highlights these elements as central to adherence promotion across healthcare settings. First, as demonstrated by Sabeti et al., verification of information and assessment of patients’ perceptions and knowledge about their illness, particularly when delivered by nurses to individuals with chronic diseases, are associated with improved adherence. This process leads to better disease management practices and encourages the adoption of recommended health behaviors [39].

Similarly, qualitative research conducted in other healthcare disciplines, such as pediatrics, has explored barriers and facilitators of comprehension and adherence among parents of children with medical complexity. These studies emphasize the importance of health literacy among patients and home caregivers in improving discharge planning and health education, findings that are aligned with the results of the present study [40]. Furthermore, an umbrella review examining factors associated with adherence to physical exercise in patients with chronic diseases and older adults identified social support, a sense of belonging, effective communication, and an active participant role as key facilitators of adherence. These elements are consistent with the findings reported by physiotherapists and psychologists in our study [41].

Overall, the findings indicate that adherence-promoting strategies vary substantially across disciplines, reflecting distinct professional frameworks and intervention priorities. Within nursing practice, adherence promotion is primarily grounded in health literacy and structured communication strategies. These results are consistent with existing evidence on adherence in patients with chronic conditions, particularly through the use of the teach-back technique. This technique has been widely documented in the literature and has shown effectiveness in improving disease understanding, patient knowledge, adherence, self-efficacy, and self-care skills among individuals with chronic illnesses [42]. Additionally, Marks et al. (2022) demonstrated the effectiveness of the teach-back method in patient education and home caregivers related to new pharmacological treatments, as it allows healthcare professionals to confirm patients’ retention and comprehension of newly provided information [43]. Importantly, this technique has also been associated with significant reductions in hospital readmissions, suggesting its potential value as a cost-effective strategy to reduce preventable rehospitalizations [44].

In contrast, physiotherapists primarily rely on motivation driven by functional achievement and social interaction, reinforcing adherence through meaningful goal attainment and group-based dynamics. In this discipline, adherence is largely facilitated by intrinsic motivation and social engagement, which is consistent with studies on physical activity interventions for chronic low back pain. These studies identified fourteen key adherence factors, among which integration into daily life, an active participant role, and social support and a sense of belonging were particularly prominent [41].

With respect to podiatry, practitioners emphasize the use of visual evidence to increase patient awareness of risks associated with asymptomatic conditions across various podiatric pathologies. There is supporting evidence for this adherence strategy, particularly among patients at risk of diabetic foot complications, where motivational interviewing and targeted communication strategies have been shown to enhance engagement in daily clinical practice [45,46]. Nevertheless, further research is needed to examine adherence strategies in podiatric care beyond disease-specific contexts, in order to develop a more comprehensive understanding of adherence among patients treated by podiatrists.

Finally, psychological interventions focus on expectation-setting and the promotion of patient autonomy, transferring responsibility for the therapeutic process to stimulate intrinsic motivation. Psychologists play a crucial role in developing behavioral skills that support effective self-management [47,48]. Moreover, the systematic use of digital reminders and technological tools has become increasingly relevant, particularly following the COVID-19 pandemic, which accelerated the adoption of digital health solutions to maintain patient contact, facilitate follow-up, and enhance long-term adherence to therapeutic intervention [49].

In summary, these findings highlight the importance of adopting a multidisciplinary and patient-centered approach to adherence promotion, in which communication strategies, motivational processes, and contextual factors are adapted to the specific professional role and patient needs to optimize long-term engagement in care.

### 4.3. Resilience and Adherence

Beyond the factors and strategies described above, the professionals interviewed consistently highlighted resilience as a key element shaping patients’ adaptive ability to sustain adherence over time. Resilience has been conceptualized as a dynamic construct reflecting the capacity for positive adaptation in the face of adversity rather than a stable personality trait [50]. Consistent with this perspective, the present findings position resilience as a key protective factor in therapeutic adherence across healthcare contexts. Participants described resilience as an adaptive resource that supports sustained engagement despite difficulties, adverse effects, or delayed improvement. Previous literature has similarly associated resilience with self-care, adherence to treatment and exercise recommendations, health-related quality of life, and illness and pain perception [51]. Moreover, resilience may predict physical functioning even more strongly than disease severity itself, partly through self-efficacy [50].

Resilience may also moderate the influence of patient expectations on adherence, particularly when expected and actual recovery trajectories diverge over time. Patient expectations significantly influence outcomes in rehabilitation and psychotherapy, including pain evolution and treatment effects [52,53]. Expectations encompass not only anticipated outcomes but also perceptions of the therapeutic process and its temporal course [54]. In line with the present findings, resilience may facilitate treatment continuity when improvement is not immediate. These findings may also reflect a broader “culture of immediacy”, characterized by expectations of rapid improvement and low tolerance for delayed therapeutic progress.

Therapeutic adherence should therefore be understood as a dynamic process shaped by psychological and contextual factors, rather than as a purely clinical outcome [1]. In this sense, adherence appears closely linked to an active patient role, including sustained engagement with treatment demands over time. Overall, the findings suggest that adherence in clinical contexts results from the interaction between patient expectations, resilience, and active engagement in the therapeutic process.

### 4.4. Clinical and Practical Implications

The findings of this study have direct implications for clinical practice, revealing that therapeutic adherence is not a generic phenomenon but a challenge shaped by the professional, sectoral, and experiential context in which each discipline operates and requires tailored responses grounded in the nature of each profession’s interventions, patient contact patterns, and clinical objectives. Nevertheless, a key finding is the central role of the therapeutic relationship and communication skills as enablers of adherence across all disciplines, underscoring the importance of incorporating training in motivational interviewing, empathic communication, and alliance-building into undergraduate and postgraduate curricula, moving beyond purely technical training.

The discipline-specific strategies identified provide concrete, practice-ready tools that can be implemented without requiring additional resources, including those traditionally associated with other disciplines. This cross-disciplinary learning potential, combined with structured communication and care coordination, could enhance the consistency of adherence-support messages and reduce the fragmentation that patients with chronic conditions frequently experience.

The role of patient resilience, perceived across all four professional groups, represents a shared opportunity to integrate resilience-informed approaches into clinical encounters, incorporating strategies such as positive reinforcement, shared decision-making, and psychoeducation to strengthen patients’ capacity to sustain treatment over time.

Finally, the systemic barriers identified, particularly limited consultation time, insufficient interdisciplinary coordination, and resource constraints, call for organizational responses beyond individual practitioners. Healthcare managers and policymakers should consider these findings when designing care models, allocating resources, and evaluating the structural conditions that support therapeutic adherence in clinical practice.

### 4.5. Methodological Considerations: Strengths and Limitations

This study has several strengths worth acknowledging. The use of a qualitative design with semi-structured interviews allowed for an in-depth exploration of professionals’ perspectives in their own clinical contexts, capturing nuances that quantitative methods would not have revealed. The purposive sampling strategy achieved the inclusion of diverse settings across non-physician healthcare disciplines, enhancing the diversity and transferability of the findings, and represents an important contribution to a literature dominated by physician and pharmacist perspectives.

However, several limitations must be considered when interpreting these findings. First, the study was conducted exclusively within a national framework (Spain), which may limit the transferability of findings to other geographical, cultural, or healthcare system contexts. Second, recruitment through professional networks and the predominance of private practice settings, particularly in podiatry and psychology, may have introduced a selection bias, overrepresenting more motivated or reflective practitioners and limiting the representativeness of the public healthcare system. However, this pattern only reflects the professional landscape in this country. Discipline, sector, and years of experience are structurally intertwined in this sample, and the qualitative design does not allow these factors to be statistically disentangled. Finally, interviews were conducted within each discipline by peers, which may have introduced a shared-perspective bias and limited the capture of externally observed viewpoints.

## 5. Conclusions

The findings suggest that adherence is a multifactorial phenomenon in which patient-related factors, particularly motivation, health beliefs, and the quality of the therapeutic relationship, emerge as the most influential determinants. Meaningful differences were identified in how each discipline interprets and responds to adherence challenges, reflecting distinct professional frameworks that, rather than representing a limitation, highlight the complementary potential of interdisciplinary collaboration.

Resilience emerged consistently across all professional groups as a mediating factor supporting patients’ capacity to sustain treatment in the face of adversity. These findings highlight the value of resilience-informed, patient-centered interdisciplinary approaches in clinical practice and the importance of adherence-focused training in professional education.

## Figures and Tables

**Table 1:** Participant characteristics.

		Nurses	Physiotherapists	Psychologists	Podiatrists	Total
Sample		8	8	8	8	32
Sex	Male	2	3	3	2	10
	Female	6	5	5	6	22
Work experience	Years	18.3 (±15.2)	26.0 (±3.4)	8.8 (±7.3)	22.3 (±8.0)	20.6 (±11.3)
Care setting	Primary	3	1	0	0	4
	Hospital	5	2	1	0	8
	Community	0	1	2	0	3
	Private	0	4	5	8	17
Geographical setting	Urban	7	3	7	7	24
	Rural	1	3	1	1	6
	Both	0	2	0	0	2

**Table 2:** Descriptive overview of codes by discipline.

Categories	Abbreviated Code	Description of Code	NUR	PHY	PSY	POD	Total
Patient factors	Implication	Lack of motivation/unintentional non-adherence	2	2	2	3	9
	Low Education	Low health literacy and educational level	7	6	6	5	24
	Beliefs	Beliefs, stigma and shame	1	2	5	0	8
	Mental and Comorbidity	Mental health and comorbidity	1	3	2	5	11
	Attitude	Coping style and attitude towards treatment	5	7	4	8	24
	Changing habits	Difficulty changing established habits	2	4	2	7	15
	Socioeconomic context	Socioeconomic context and autonomy	3	2	6	2	13
	Age-related	Age-related factors	4	3	3	4	14
Nature of the health condition	Severity and symptom	Perceived severity and symptom visibility	2	1	2	2	7
Treatment factors	Complexity	Complexity of the treatment regimen	3	5	1	1	10
	Duration	Treatment duration	3	2	1	6	12
	Adverse Effects	Adverse effects	5	0	0	2	7
	Economic cost	Economic cost of treatment	2	2	5	2	11
	Lack personalization	Lack of treatment personalization	3	5	4	2	14
Healthcare system factors	Limited time	Limited consultation time	5	1	5	1	12
	Continuity	Continuity of care	5	1	8	4	18
	Lack resources	Insufficient resources, access and waiting lists	5	7	4	6	22
	Coordination	Care coordination and interdisciplinary support	5	1	7	4	17
Professional factors	Attitude	Communication and empathic attitude	7	8	8	7	30
	Communications skills	Motivational interviewing skills	2	3	0	0	5
	Therapeutic relationship	Trust and therapeutic relationship	8	7	8	8	31

Abbreviations: NUR: Nurses; PHY: Physiotherapists; PSY: Psychologists; POD: Podiatrists. Note: Frequencies represent the number of interviews in which each code was identified at least once, not the total number of coded text segments.

**Table 3:** Strategies used to improve adherence.

Strategies	Definition	Activities to Improve Adherence
Health education and information	Ensures the patient understands the “why” behind the treatment	▪ Teach-back technique: asking the patient to explain the treatment as if they were the professional to verify comprehension. ▪ Detailed pathophysiological explanation: investing time to explain the relationship between symptoms and the diagnosis ▪ Visual aids and infographics: using brochures, hand-drawn diagrams, or videos. ▪ Digital literacy: teaching patients to use reliable health websites or how to cross-reference AI-generated information.
Treatment simplification and personalization	Adapting protocols to the patient’s daily life to reduce therapeutic burden	▪ Regimen simplification: reducing the number of exercises/doses and using plain language. ▪ Personalized roadmap: a written document with exact steps and contact information. ▪ Functional goal consensus: aligning goals with activities the patient values (e.g., dancing or walking a pet).
Family and caregiver involvement	Engaging the patient’s social environment as a fundamental ally.	▪ Caregiver training: essential for dependent patients. ▪ Family mediation: helping resolve domestic conflicts that may hinder care.
Technology and reminders	Utilizing digital tools to maintain contact and prevent forgetfulness	▪ SMS/WhatsApp reminders: text messages sent 24–48 h before appointments. ▪ Mobile apps: for habit tracking, video-guided exercises, or medication alarms. ▪ Telehealth: remote follow-ups to reduce the need for travel ▪ Virtual reality and QR codes: for phobia desensitization or accessing home exercise protocols.
Psychological, motivational and relational strategies	Focused on patient attitude and the strength of the therapeutic alliance	▪ Positive reinforcement: celebrating short-term achievements to maintain motivation. ▪ Gamification and group activities: using humor and community settings to normalize clinical experience. ▪ Direct confrontation: honest, serious communication when a lack of collaboration requires an attitude shift. ▪ Empowerment: returning autonomy to the patient so they take responsibility for their process. ▪ Motivational metaphors: using analogies (e.g., Gulliver Syndrome) to explain cumulative risks.
Administrative and appointment management	Organizational changes to facilitate continuity of care	▪ Flexible scheduling: ensuring the next visit is booked before departure, adapted to the patient’s schedule. ▪ Brief follow-ups: “Check-in” phone calls to monitor progress without requiring a formal visit. ▪ Strategic appointment spacing: gradually increasing the time between sessions as the patient improves to foster independence.

## Data Availability

The original contributions presented in this study are included in the article. Further inquiries can be directed to the corresponding author. The full interview transcripts contain potentially identifiable information and are subject to confidentiality and ethical restrictions established during the informed consent process. Therefore, the complete dataset cannot be made publicly available.
